# Human chromosome 3p21.3 carries *TERT* transcriptional regulators in pancreatic cancer

**DOI:** 10.1038/s41598-021-94711-6

**Published:** 2021-07-28

**Authors:** Takuki Yagyu, Takahito Ohira, Ryutaro Shimizu, Masaki Morimoto, Yuki Murakami, Takehiko Hanaki, Kyoichi Kihara, Tomoyuki Matsunaga, Manabu Yamamoto, Naruo Tokuyasu, Teruhisa Sakamoto, Yoshiyuki Fujiwara, Hiroyuki Kugoh

**Affiliations:** 1 Division of Gastrointestinal and Pediatric Surgery, Department of Surgery, School of Medicine, Faculty of Medicine, Tottori University, Yonago, Tottori Japan; 2 Department of Molecular and Cellular Biology, Division of Genome and Cellular Function, Tottori University, Yonago, Tottori Japan; 3 Chromosome Engineering Research Center, Tottori University, Yonago, Tottori Japan; 4 Division of Urology, Department of Surgery, Faculty of Medicine, Tottori University, Yonago, Tottori Japan

**Keywords:** Cancer, Gastrointestinal cancer

## Abstract

Frequent loss of heterozygosity (LOH) on the short arm of human chromosome 3 (3p) region has been found in pancreatic cancer (PC), which suggests the likely presence of tumor suppressor genes in this region. However, the functional significance of LOH in this region in the development of PC has not been clearly defined. The human telomerase reverse transcriptase gene (*hTERT)* contributes to unlimited proliferative and tumorigenicity of malignant tumors. We previously demonstrated that *hTERT* expression was suppressed by the introduction of human chromosome 3 in several cancer cell lines. To examine the functional role of putative *TERT* suppressor genes on chromosome 3 in PC, we introduced an intact human chromosome 3 into the human PK9 and murine LTPA PC cell lines using microcell-mediated chromosome transfer. PK9 microcell hybrids with an introduced human chromosome 3 showed significant morphological changes and rapid growth arrest. Intriguingly, microcell hybrid clones of LTPA cells with an introduced human chromosome 3 (LTPA#3) showed suppression of *mTert* transcription, cell proliferation, and invasion compared with LTPA#4 cells containing human chromosome 4 and parental LTPA cells. Additionally, the promoter activity of *mTert* was downregulated in LTPA#3. Furthermore, we confirmed that *TERT* regulatory gene(s) are present in the 3p21.3 region by transfer of truncated chromosomes at arbitrary regions. These results provide important information on the functional significance of the LOH at 3p for development and progression of PC.

## Introduction

Pancreatic cancer (PC) is a lethal malignancy with a poor prognosis (5-year survival rate of < 10%)^1^. The lethality is attributed to late diagnosis, early metastasis, and resistance to chemotherapy^2^. Compared with gemcitabine alone, the chemotherapeutic regimen comprising 5-fluorouracil, leucovorin, irinotecan, and oxaliplatin has improved the median overall survival from 6.8 months to 11.1 months in patients with untreated metastatic PC^3^. However, the tolerability remains a concern because of the high systemic toxicity of these drugs. Additionally, unlike other cancers, there are few effective molecularly targeted drugs for PC, and only a small number of patients will benefit from them^4^. The molecular mechanism involved in PC progression remains unclear, and further research is urgently required to improve diagnosis and develop effective treatments for PC.

Multiple tumor suppressor genes (TSGs) have been identified by positional cloning and analysis of loss of heterozygosity (LOH)^5–8^. In PC, frequent LOH has been detected at several chromosomal locations, including the 3p, 6q, 8p, 9p, 17p, and 18q regions^9,10^. The 9p, 17p, and 18q regions have particularly high frequencies of LOH (> 80%) and encode cyclin dependent kinase inhibitor 2A, tumor protein 53, and SMAD family member 4 (also known as deleted in pancreatic carcinoma 4), respectively. These genes are frequently mutated or deleted in PC, which suggests a close relationship between LOH and mutation of TSGs. Although LOH of the 3p region has also been observed with high frequency (75%), specific TSGs involved in the development or progression of PC have yet to be identified. Therefore, the discovery of TSGs in the 3p region may facilitate our understanding of the molecular mechanisms that are involved in PC development. Additionally, the frequent LOH of the 3p region has been observed in several other cancers, such as renal cell carcinoma (RCC), non-small cell lung cancer, and oral squamous cell carcinoma (OSCC)^11–13^. Thus, this chromosomal region may play a crucial role in the development of malignant tumors.

Telomerase is an enzyme that maintains telomere length and confers unlimited proliferative potential and tumorigenicity to cancer^14^. The activity of telomerase is primarily attributed to the expression of telomerase reverse transcriptase (*TERT*)^15^. Previous studies have demonstrated that *TERT* expression is not only associated with replicative immortality, but also with cancer cell invasion, metastasis, and induction of a stem cell-like phenotype^16–18^. Telomerase is activated in most cancer cells, including PC, while its expression is suppressed in normal somatic cells^19^. Therefore, telomerase is an ideal target for novel anticancer drugs and diagnostic agents; however, it has not yet been widely used in clinical settings. We have shown that telomerase activity was suppressed in RCC and OSCC cell lines after the introduction of normal human chromosome 3 using the chromosome engineering technique microcell-mediated chromosome transfer (MMCT)^20,21^. We identified 3p21.3 as the telomerase inhibitory region in the RCC cells by transferring truncated chromosomes that had deletions from the 3p22 or 3p21.3 locus to the terminal end of the short arm^20^.

*TERT* expression is strictly regulated by multiple transcriptional activators and repressors, such as the c-MYC oncogene protein, specificity protein 1, hypoxia-inducible factor 1, E2F transcription factor 1, mitotic arrest deficient 1 protein, multiple endocrine neoplasia type 1 protein, Wilms’ tumor protein 1, retinoblastoma protein, and paired-like homeodomain 1 (PITX1) protein, although the exact regulatory mechanism has not been completely elucidated^22,23^. A precise understanding of the mechanism by which *TERT* expression is repressed in somatic cells will help clarify the process of carcinogenesis. Furthermore, the identification of *TERT*-regulated factors may lead to the development of novel therapeutic and diagnostic approaches, as well as reveal telomerase-induced oncogenic pathways.

By using unique chromosome engineering techniques, this study demonstrated that the *TERT* suppressor in PC is located at the 3p21.3 region. Furthermore, we verified that *TERT* expression was repressed by decreasing promoter activity. Our results may provide important information regarding the functional significance of the LOH at the 3p region in pancreatic carcinogenesis and may lead to the identification of novel TSGs encoded by the 3p21.3 locus.

## Results

### PK9 microcell hybrids with human chromosome 3 could not be established because of arrested cell proliferation

**Figure 1:**
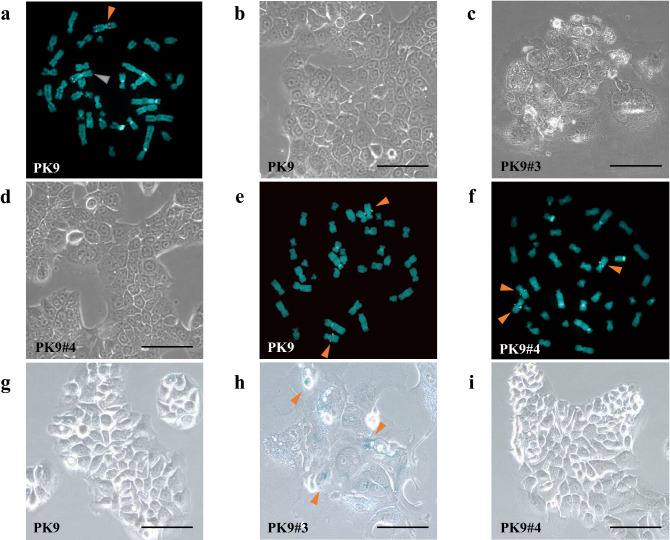
PK9 cells have monoallelic deletion at the 3p21.3 region, and PK9 microcell hybrids with human chromosome 3 could not be established due to arrested cell proliferation. (**a**) Fluorescence in situ hybridization (FISH) analysis of human pancreatic cancer PK9 cells in metaphase. Rhodamine-labeled RP-6 234N4 pPAC4 clone containing the human chromosome 3p21.3 genomic DNA and FITC-labeled RP11-82C9 BAC that included the 3q26.2 region were used as probe DNA. The orange arrowheads indicate a normal human chromosome 3 which has both rhodamine and FITC signals. The gray arrowhead points out an abnormal chromosome 3 with only the FITC signal. The morphology of (**b**) parental PK9 cells and (**c**) G418-resistant PK9 microcell hybrids with human chromosome 3 (PK9#3). Scale bar 100 µm. FISH analysis of (**d**) PK9 cells and (**e**) PK9#4 cells. FITC-labeled RP11-84C13 BAC clone that included the 4q22.1 region was used as probe DNA. The arrowhead indicates parental and transferred human chromosome 4. (**f**) The morphology of PK9#4 cells. Scale bar 100 µm. Images of β-galactosidase staining of (**g**) parental PK9 cells, (**h**) PK9#3 cells, and (**i**) PK9#4 cells. The arrowhead indicates representative SA-β-gal positive cells. Scale bar 100 µm.

To investigate the status of chromosome 3 in PK9 cells, we performed FISH analysis using PAC and BAC probes that contained the genomic DNA from the 3p21.3 (rhodamine) and 3q26.2 (FITC) chromosomal regions, respectively. Although PK9 cells had two copies of chromosome 3, one displayed chromosomal abnormalities that included the deletion of the 3p21.3 region (Fig. 1a).

We transferred human chromosome 3 tagged with *pSV2neo* into PK9 cells using MMCT to determine whether chromosome 3 induced tumor suppressive effects in human PC. However, G418-resistant PK9 microcell hybrids with human chromosome 3 (PK9#3) displayed significant morphological changes and arrested cell proliferation after several population divisions compared with parental PK9 cells. The colonies showed an expanded cytoplasm that was similar to senescent cells and survived for approximately one month after proliferation ceased (Fig. 1b,c). To confirm whether this phenomenon was caused by the introduction of chromosome 3, we also transferred chromosome 4 into PK9 cells. Microcell hybrids with an introduced chromosome 4 (PK9#4) were easily established and exhibited a morphology and rapid proliferation that was similar to the parental PK9 cells (Fig. 1d). The presence of transferred chromosome 4 in each hybrid clone was confirmed by FISH analysis using a BAC probe (FITC) containing the 4q22.1 genomic DNA region. The parental PK9 cells had two copies of chromosome 4, whereas all clones of PK9#4 had three copies of chromosome 4, which confirmed the presence of the transferred chromosome (Fig. 1e,f).

It has been reported that cellular senescence was significantly increased by knockdown of *hTERT* in gastric cancer cells^24^. Furthermore, pancreatic cancer cell lines including PK9 cells have significantly shorter telomeres than normal pancreas^25^. These evidences suggested that it may be possible to induction of cellular senescence at early stage of cell division in some cancer cells that have short telomeres. To further explore the cause of the phenomenon that exhibited cell growth arrest by the introduction of human chromosome 3 into PK9 cells, we analyzed cellular senescence using the SA-β-gal assay. Cellular senescence significantly increased in PK9#3 cells compared to parental and PK9#4 cells (Fig. 1g,h,i) may suggest that the tumor suppressor gene(s) on chromosome 3 regulate *hTERT* transcription and/or cell proliferation.

### Suppression of *mTert* expression by introduction of an intact human chromosome 3 into mouse LTPA cells

**Figure 2:**
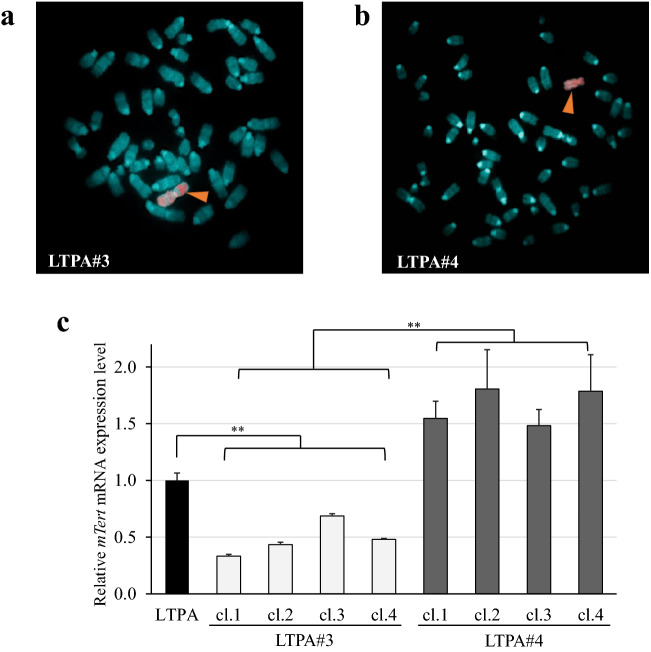
Suppression of *mTert* expression by introduction of an intact human chromosome 3 into murine LTPA cells. Fluorescence in situ hybridization (FISH) analysis of (**a**) LTPA#3 cells and (**b**) LTPA#4 cells. Rhodamine-labeled human COT-1 DNA was used as probe DNA. The arrowhead indicates a transferred human chromosome 3 or 4. (**c**) Quantitative RT-PCR analysis of relative *mTert* mRNA expression levels in LTPA, LTPA#3, and LTPA#4 cells. The mRNA expression of *mGapdh* was used as the internal control. Data are presented as means ± S.E. of three independent experiments (**P < 0.001).

We examined whether the tumor suppressor region on chromosome 3 functioned in the murine LTPA pancreatic cancer line and whether the tumor suppressive mechanism was related to telomerase suppression. The microcell hybrid clones with an introduced human chromosome 3 (LTPA#3) and 4 (LTPA#4) were obtained by MMCT, and the presence of the each transferred human chromosome was confirmed by FISH analysis using human COT-1 DNA as a probe (rhodamine) (Fig. 2a,b).

We analyzed the expression of *mTert* mRNA in the LTPA#3 and LTPA#4 hybrid cells by using qRT-PCR to determine whether *TERT* repressor gene(s) on human chromosome 3 might function in mouse PC cells. The LTPA#3 microcell hybrid cells exhibited significant transcriptional suppression of *mTert* compared with the parental LTPA and LTPA#4 hybrid cells (Fig. 2c). Thus, these results suggest that a tumor suppressor gene or genes on chromosome 3 control *mTert* transcription, and, therefore, may play a crucial role in the development and progression of PC.

### Introduction of human chromosome 3 in LTPA cells inhibits cell proliferation and invasion

**Figure 3:**
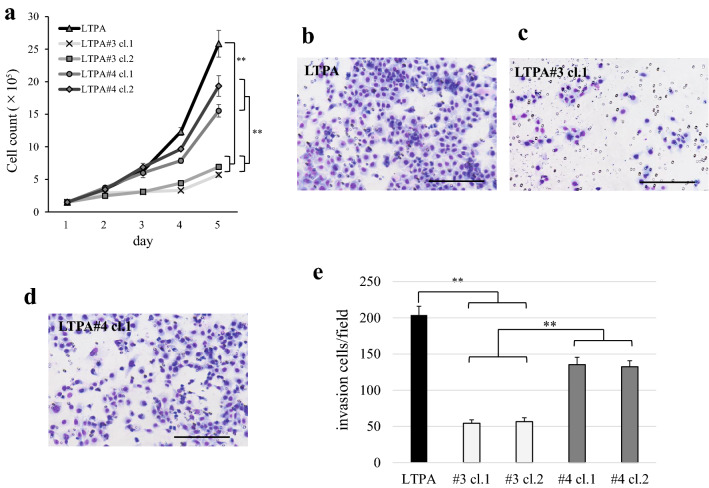
Induction of human chromosome 3 into LTPA cells inhibits cell proliferation and invasion. (**a**) Cell numbers for LTPA, LTPA#3, and LTPA#4 cells over a 5 day period. The bars correspond to means ± S.E. of three independent experiments (**P < 0.001). Representative images of the invasion assay for (**b**) LTPA, (**c**) LTPA#3, and (**d**) LTPA#4 cells. Scale bar 200 µm. (**e**) Counts of invading cells per five microscopic fields for LTPA, LTPA#3, and LTPA#4 cells. The bars correspond to means ± S.E. of three independent experiments (**P < 0.001).

Next, we examined whether transfer of human chromosome 3 into LTPA cells affected cell proliferation similar to PK9 cells. Proliferation of LTPA#3 microcell hybrid cells was significantly decreased compared with parental LTPA and LTPA#4 cells (Fig. 3a). Additionally, we examined cell invasion potential in parental LTPA, LTPA#3, and LTPA#4 cells because recent findings have indicated that a relationship exists between telomerase activity and cancer cell invasion and metastasis^17^. The invasion assays showed a significant decrease in the number of invading LTPA#3 cells compared with the other cells (Fig. 3b–e). These results showed that suppression of *mTert* gene expression by chromosome 3 was accompanied by the suppressive effects on cell proliferation and invasion in PC.

### Confirmation of an *mTert* suppressor region on 3p21.3 after transfer of truncated chromosomes

**Figure 4:**
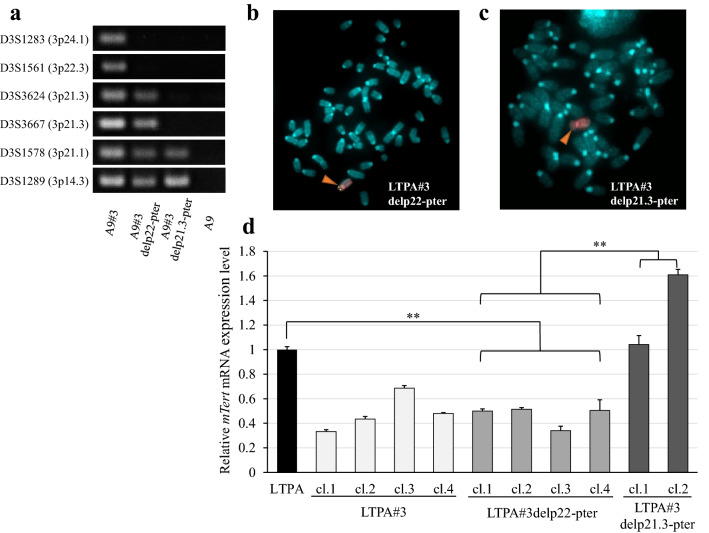
Identification of an *mTert* suppressor region on 3p21.3 by transfer of truncated chromosomes. (**a**) PCR analysis of truncated chromosomes in A9 cells using STS markers for chromosome 3. Full-length gels of the PCR analysis are presented in Supplementary Fig. S1. Fluorescence in situ hybridization analysis of (**b**) LTPA#3delp22-pter and (**c**) LTPA#3delp21.3-pter cells. FITC-labeled RP-6 234N4 pPAC4 that included the 3p21.3 region and rhodamine-labeled human COT-1 DNA were used as probe DNA. The arrowhead indicates a transferred truncated human chromosome 3. (**d**) Quantitative RT-PCR analysis of relative *mTert* mRNA expression levels in LTPA, LTPA#3, LTPA#3delp22-pter, and LTPA#3delp21.3-pter cells. The mRNA expression of *mGapdh* was used as the internal control. Data are presented as means ± S.E. of three independent experiments (**P < 0.001).

To determine whether the 3p21.3 region carries the *TERT* repressor element in PC, we truncated chromosome 3 by deleting the regions from the 3p22 locus to the telomere of the short arm (#3delp22-pter) and the 3p21.3 locus to the telomere of the short arm (#3delp21.3-pter) as previously described^20^. PCR analysis using six STS markers revealed that the specific target regions were deleted on each of the two truncated chromosomes in the mouse A9 microcell hybrid cells (Fig. 4a, Supplementary Fig. S1 online). The truncated chromosomes were transferred independently into LTPA cells by MMCT. The transfer of truncated chromosomes in the isolated LTPA microcell hybrids LTPA#3delp22-pter and LTPA#3delp21.3-pter was confirmed using FISH analyses with a human COT-1 DNA probe (rhodamine) and PAC probe containing the 3p21.3 genomic DNA region (FITC), respectively (Fig. 4b,c).

We analyzed the expression of *mTert* mRNA in the LTPA#3delp22-pter and LTPA#3delp21.3-pter microcell hybrids by using qRT-PCR. The expression of *mTert* was repressed in LTPA#3delp22-pter and LTPA#3 cells compared with parental cells. Conversely, high expression of *mTert* was maintained in the LTPA#3delp21.3-pter cells without any changes in cell growth properties (Fig. 4d).

We transferred the truncated chromosome 3 with deleted 3p21.3-pter into PK9 cells that had shown rapid growth arrest by the introduction of intact chromosome 3. The isolated microcell hybrid cells (PK9#3delp21.3-pter) showed no morphological changes, unlike after the transfer of chromosome 3 (see Supplementary Fig. S2 online). Supplementary Table S1 (see online) shows the transfer efficiency of chromosomes 3, 4, and 3delp21.3-pter in PK9 cells. MMCT of chromosome 3 was performed six times more than that of the control, but no clone could be established because of cell growth arrest. These results may support the hypothesis that the 3p21.3 region carries various TSGs that are involved in regulating cell growth arrest and *TERT* repression in PC.

### The *mTert* repressive effect of the gene(s) located on human chromosome 3 is due to regulation of *mTert* promoter activity

**Figure 5:**
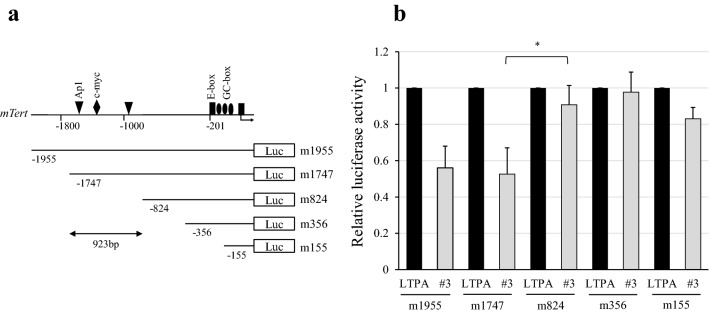
The *mTert* repressive effect of the gene(s) on human chromosome 3 is due to the regulation of *mTert* promoter activity. (**a**) The schema shows the truncation sites of the *mTert* promoter for each of the luciferase (Luc) reporter plasmids. (**b**) The luciferase activity was standardized using *Renilla reniformis* luciferase activity from a co-transfected pGL4.70 plasmid. Data are presented as means ± S.E. of three independent experiments (*P = 0.02).

The mechanism of increased *TERT* expression in various cancers is generally known to result from transcription factor activation, epigenetic modifications, and mutations of the *TERT* promoter^26^. However, it has been reported that PC has a low frequency of mutations in the *TERT* promoter^27^. Therefore, we hypothesized that the repression of *mTert* expression by human chromosome 3 may be due to modulation of *mTert* promoter activity by transcription factors. To verify this hypothesis, we performed a reporter assay using an *mTert* promoter-luciferase reporter plasmid (pGL3). The reporter plasmids were constructed carrying the full length of the *mTert* promoter region (m1955) and promoter regions with deletions in the 5ʹ end (m1747, m 824, m356, and m155) (Fig. 5a)^23^. These pGL3-mTert-Luc reporters were transfected into parental LTPA and LTPA#3 cells, and the effect on the transcriptional activity of the *mTert* promoter was examined by measuring luciferase activity. The transfection of the m1955 and m1747 *mTert* promoter regions revealed reduced luciferase activity in LTPA#3 cells compared with LTPA cells. However, luciferase activity was not decreased in LTPA#3 cells transfected with the m824, m356, and m155 promoter regions (Fig. 5b). The results of these reporter assays suggest that the mechanism of repression of *mTert* expression by human chromosome 3 is due to reduced promoter activity via trans-factors and that the 923-bp sequence between − 1747 and − 824 in the *mTert* promoter region is critical for the control of *TERT* transcription.

## Discussion

We have previously clarified that human chromosome 3 carries factors that control *TERT* transcription in RCC and OSCC^20,21^. Although a high frequency of LOH on the short arm of chromosome 3 has been observed in PC, the function of TSGs in this region remains unclear. To our knowledge, this is the first report referring to the localization of telomerase suppressor genes at the 3p21.3 chromosomal region in PC.

A theory of multistage carcinogenesis has been proposed in which PC originates from pancreatic ductal epithelial cells and progresses to cancer via pancreatic intraepithelial neoplasia (PanIN), a precancerous lesion^28^. Although telomerase is active in PC, it is known that telomere length is shortened in the first stage of carcinogenesis^29,30^. Telomere shortening can lead to aberrant fusion of chromosomal ends, causing chromosomal instability and promoting further progression of the neoplasm^31^. Furthermore, these unstable chromosomes may undergo the breakage-fusion-bridge cycle, which results in loss of function of TSGs and gain of function of oncogenes^28,31,32^. Telomere length continues to shorten during advancing atypia of PanIN^30,33^. The precancerous lesions that have reached the limit of telomere shortening may be in crisis, which leads to immortalization by releasing the previously suppressed expression of *TERT*. Therefore, identification of a novel telomerase repressor gene(s) on the 3p21.3 chromosomal region may facilitate our understanding of the molecular mechanisms in the multistage carcinogenesis model for PC, especially in the late stages.

In this study, we used both human PK9 and murine LTPA pancreatic cancer cells. The suppressive effects on cell growth properties and *mTert* expression induced by the introduction of human chromosome 3 into LTPA cells were not observed after the introduction of the truncated chromosome #3delp21.3-pter, which does not have the 3p21.3 region. The rapid cell growth arrest after the introduction of chromosome 3 was greater in PK9 cells than in LTPA cells. It is possible that human chromosome 3 may carry specific cell growth regulators that are more functional in human cells than mouse cells. The difference in cell growth suppression may also be affected by the telomere length in humans and mice. Humans and mice have telomere DNA lengths of 5–10 kb and 30–40 kb, respectively^34^. In general, inhibition of telomerase has stronger anticancer effects in cells with shorter telomere lengths^35,36^. These findings support the present results that showed that the growth of PK9 cells, which have a shorter telomere length, was robustly arrested by the introduction of human chromosome 3 compared with LTPA cells. Moreover, a study based on human tissues collected in a clinical setting showed that the telomere length of PC cells is approximately one-third of that found in normal pancreatic ductal epithelial cells^30^. In brief, these previous reports and our results suggest that telomerase suppression has potential as an attractive therapeutic target in human PC, which has shortened lengths of telomere DNA.

There are various TSGs (*AXUD1, HYAL2, FUS1, LIMD1, NPRL2, CACNA2D2* and *RASSF1*) in 7-Mb of the 3p21.3 region^37–39^. Among them, the Ras-association domain family-1 (*RASSF1*) gene is a major tumor suppressor gene that inhibits the RAS pathway, which is upregulated in the majority of PCs^40,41^. However, it is unlikely that *RASSF1* is a *TERT* repressor gene because the forced-expression of *RASSF1* in the RCC cell line RCC23 did not repress *TERT* promoter activity^42^.

In our study, the results of the reporter assays may be useful for selecting candidate genes. The reporter assays suggested that the 923-bp sequence between − 1747 and − 824 bp of the *mTert* promoter was a candidate site for transcription factor binding. The 923-bp sequence may help to identify telomerase suppressor genes by comparing the sequence with putative transcription factor binding sites. Chromosome 3 may carry elements for direct or indirect negative transcriptional regulation of *TERT* expression. Another consideration for candidate genes in the 3p21.3 region is that multiple TSGs may interact to repress *TERT*, rather than a single gene. We have previously shown that at least two *hTERT* negative regulators may be present on human chromosome 3 by using whole cell fusion of human squamous carcinoma cells HSC3 and RCC23 cells^21^. Deletions in the 3p21.3 region are frequent events in several cancers, and this region is considered to be a TSG cluster because multiple TSGs are densely localized to this chromosomal segment^37^. Most studies on the 3p21.3 region have focused on the anticancer function of each single gene^38,40^. However, TSGs are frequently deleted through loss of whole chromosomes or large chromosomal segments. Recent studies have shown that TSGs can cooperate with many nearby genes that are deleted together, which results in a more invasive disease^43^. We have previously reported that *PITX1*, a negative regulator of *hTERT*, interacts with zinc finger CCHC-type containing 10 (*ZCCHC10*) to regulate *hTERT* transcription, and both genes are encoded together in the 5q31.1 region^44^. Therefore, chromosome engineering technologies that can transfer an entire chromosome or chromosomal fragments, which contain the essential genomic context for regulation of gene transcription, may enable elucidation of tumor suppressor functions by specific chromosomal domains^45^.

In conclusion, the *TERT* repressor gene(s) was found to be localized within the 3p21.3 chromosomal region in PC. Moreover, the gene(s) may regulate *TERT* promoter activity. This study provides important information and materials for identification of novel *TERT* suppression genes on human chromosome 3p21.3 by an approach that uses a database of transcription factor binding profiles. Additionally, we previously identified *PITX1* as a novel telomerase repressor gene on human chromosome 5 that directly regulated *hTERT* transcription. This was accomplished by using comparative microarray analyses of microcell hybrid clones comprising mouse B16F10 melanoma cells with an introduced human chromosome 5^23^. Using a similar approach, it may be possible to identify the *TERT* repressor gene(s) on 3p21.3. The identification and characterization of putative *TERT* repressor genes on the 3p21.3 region should facilitate our understanding of the molecular mechanisms involved in the development of PC.

## Materials and methods

### Cell culture

Mouse A9 was purchased from the American Type Culture Collection (ATCC) (Manassas, VA), and A9 microcell hybrid cells containing chromosomes were established in our laboratory^20,45^. A9 microcell hybrid cells containing human chromosomes were cultured in Dulbecco’s modified Eagle’s medium (DMEM; Sigma-Aldrich, St. Louis, MO, USA) supplemented with 10% fetal bovine serum (FBS; HyClone, Logan, UT, USA) and 800 µg/mL G418 (Calbiochem, La Jolla, CA, USA). The human PK9 and murine LTPA pancreatic cancer cell lines were cultured in DMEM containing 10% FBS and used as recipient cells for microcell fusion. PK9 cells were obtained from the Cell Resource Center of Biomedical Research Institute of Development, Aging and Cancer at Tohoku University. LTPA cells were purchased from ATCC. PK9 and LTPA microcell hybrid cells containing intact or truncated chromosomes were selected with 400 and 600 µg/mL G418, respectively. All cells were cultured at 37℃ in a humidified incubator with 5% CO_2_. In this study, we utilized only cell lines and no tissue samples obtained from human/animal.

### Microcell-mediated chromosome transfer

MMCT was performed as previously described^46^. Briefly, the A9 donor cells were incubated with 0.05 µg/mL colcemid (Sigma-Aldrich) in DMEM containing 20% FBS for 48 h. Micronuclei were harvested by treatment with 10 mg/mL cytochalasin B and centrifugation and were sequentially filtered through 8-, 5-, and 3-mm polycarbonate filters (Nuclepore, UK). Fusion was mediated by the addition of 47% polyethylene-glycol 1000, followed by extensive washing with serum-free DMEM. After incubation for 24 h in DMEM supplemented with 10% FBS, the cells with transferred chromosomes were selected in the presence of G418 as described above.

### Fluorescence in situ hybridization (FISH)

To confirm the stable transfer of chromosomes, FISH was performed using cells in metaphase. RP-6 234N4 PAC, RP11-82C9 BAC, and RP11-84C13 BAC DNA contained the genomic DNA from the 3p21.3, 3q26.2, and 4q22.1 chromosomal regions, respectively, and were used as probe DNA. Human COT-1 DNA was used for detection of the human chromosomes in mouse LTPA cells. Each DNA preparation was labeled with digoxigenin-11-dUTP or with biotin-16-dUTP using a nick translation kit (Roche Diagnostics, Germany). The preparation of chromosomes and the probe, hybridization, washing, and signal detection were performed as previously described^46^.

### Senescence-associated β-galactosidase activity

We examined senescence-associated β-galactosidase activity using the SA-β-gal Staining Cellular Senescence Assay Kit (Cell Biolabs Inc., San Diego, CA) according to the manufacturer's protocol.

### Quantitative RT-PCR

RNA isolation and reverse transcriptase (RT)-PCR was performed as described previously^21^. The primers 5ʹ-ATGTCACGGAGAGCACATTC-3ʹ and 5ʹ-CTGCAGATGGGCATGGCTA-3ʹ were used for the detection of *mTert* expression in the mouse LTPA cells. As an internal control, *mGapdh* expression was examined with the primers 5ʹ-TCATTGTCATACCAGGAAATGAGC-3ʹ and 5ʹ-GTCTCCTGCGACTTCAACAG-3ʹ.

### Cell proliferation assay

LTPA cells and LTPA hybrid cells containing chromosome 3 or 4 were seeded in 60-mm cell culture dishes at a concentration of 1.5 × 10^5^ cells/dish. Cells were counted each day using a Coulter Counter Z2 (Beckman Coulter, Woerden, the Netherlands) and the average cell number for three wells was calculated. The experiments were performed at least three times.

### Cell invasion assay

Cell invasion assays were performed using BD BioCoat™ Matrigel™ invasion chambers with a membrane pore size of 8.0 µm (BD Biosciences, Bedford, MA, USA) following the manufacturer's protocol. The cells were starved in serum-free medium for 48 h and seeded in the upper inserts at a final concentration of 5.0 × 10^4^ cells/well. The lower chambers were filled with medium containing 10% FBS. After 48 h, non-invading cells were removed from the top of the filter with a cotton swab. The invading cells at the bottom of the filter were stained with Diff-Quik stain (Siemens Healthcare Diagnostics) and counted using five different microscopic fields of view at a magnification of 200 ×. Each of the experiments was performed at least three times.

### Genomic PCR analysis

The presence or absence of specific regions on human chromosome 3 were confirmed by PCR using six specific sequence-tagged site (STS) markers (D3S1283, D3S1561, D3S3624, D3S3667, D3S1578, and D3S1589). Primer information was obtained from the National Center for Biotechnology Information database, U.S. National Library of Medicine. PCR was performed using 35 cycles comprising 30 s at 94 ℃, 30 s at 55–60 ℃, and 30 s at 68 ℃.

### Plasmid construction and luciferase assay

Various lengths of the *mTert* promoter region were previously described^23^. Cells were incubated in 12-well plates 24 h prior to transfection. After 24 h, 0.1 µg of each reporter plasmid was co-transfected with 0.25 µg pGL4.70-Renilla (Promega) as an internal control using Lipofectamine® LTX Reagent (Invitrogen) in accordance with the manufacturer’s protocol. The cells were lysed 24 h after transfection and subjected to a luciferase assay using the Picagene Dual SeaPansy Luminescence Kit (TOYO INK, Tokyo, Japan) following standard protocols. All experiments were performed at least three times.

### Statistics

Data from more than three separate experiments are presented as means ± S.E. Significance was established at *P*-values less than 0.05 using an unpaired two-tailed Student’s *t* test. All statistical analyses were performed using SPSS software (SPSS for Mac Version 25; IBM Corp., Armonk, NY, USA).

## Supplementary Information

Supplementary Information.

## References

[1] Siegel RL, Miller KD, Jemal A (2020). Cancer statistics, 2020. CA Cancer J. Clin..

[2] Stromnes IM, DelGiorno KE, Greenberg PD, Hingorani SR (2014). Stromal reengineering to treat pancreas cancer. Carcinogenesis.

[3] Conroy T (2011). FOLFIRINOX versus gemcitabine for metastatic pancreatic cancer. N. Engl. J. Med..

[4] Mizrahi JD, Surana R, Valle JW, Shroff RT (2020). Pancreatic cancer. Lancet.

[5] Kinzler KW (1991). Identification of FAP locus genes from chromosome 5q21. Science.

[6] Friend SH (1986). A human DNA segment with properties of the gene that predisposes to retinoblastoma and osteosarcoma. Nature.

[7] Antony J, Zanini E, Birtley JR, Gabra H, Recchi C (2021). Emerging roles for the GPI-anchored tumor suppressor OPCML in cancers. Cancer Gene Ther..

[8] Chauffaille M, Zalcberg I, Barreto WG, Bendit I (2020). Detection of somatic TP53 mutations and 17p deletions in patients with chronic lymphocytic leukemia: a review of the current methods. Hematol. Transfus Cell Ther..

[9] Calhoun ES (2006). Identifying allelic loss and homozygous deletions in pancreatic cancer without matched normals using high-density single-nucleotide polymorphism arrays. Cancer Res..

[10] Qiu W (2011). Disruption of p16 and activation of Kras in pancreas increase ductal adenocarcinoma formation and metastasis in vivo. Oncotarget.

[11] Alimov A (2000). Combined LOH/CGH analysis proves the existence of interstitial 3p deletions in renal cell carcinoma. Oncogene.

[12] Partridge M, Emilion G, Langdon JD (1996). LOH at 3p correlates with a poor survival in oral squamous cell carcinoma. Br. J. Cancer.

[13] Zabarovsky ER, Lerman MI, Minna JD (2002). Tumor suppressor genes on chromosome 3p involved in the pathogenesis of lung and other cancers. Oncogene.

[14] Counter CM (1992). Telomere shortening associated with chromosome instability is arrested in immortal cells which express telomerase activity. EMBO J..

[15] Meyerson M (1997). hEST2, the putative human telomerase catalytic subunit gene, is up-regulated in tumor cells and during immortalization. Cell.

[16] Low KC, Tergaonkar V (2013). Telomerase: Central regulator of all of the hallmarks of cancer. Trends Biochem. Sci..

[17] Hannen R, Bartsch JW (2018). Essential roles of telomerase reverse transcriptase hTERT in cancer stemness and metastasis. FEBS Lett..

[18] Liu Z (2013). Telomerase reverse transcriptase promotes epithelial-mesenchymal transition and stem cell-like traits in cancer cells. Oncogene.

[19] Collins K, Mitchell JR (2002). Telomerase in the human organism. Oncogene.

[20] Abe S (2010). Localization of an hTERT repressor region on human chromosome 3p21.3 using chromosome engineering. Genome Integr..

[21] Nishio S (2015). Repression of hTERT transcription by the introduction of chromosome 3 into human oral squamous cell carcinoma. Biochem. Biophys. Res. Commun..

[22] Yuan X, Larsson C, Xu D (2019). Mechanisms underlying the activation of TERT transcription and telomerase activity in human cancer: Old actors and new players. Oncogene.

[23] Qi DL (2011). Identification of PITX1 as a TERT suppressor gene located on human chromosome 5. Mol Cell Biol.

[24] La SH, Kim SJ, Kang HG, Lee HW, Chun KH (2016). Ablation of human telomerase reverse transcriptase (hTERT) induces cellular senescence in gastric cancer through a galectin-3 dependent mechanism. Oncotarget.

[25] Hata T (2018). Simple detection of telomere fusions in pancreatic cancer, intraductal papillary mucinous neoplasm, and pancreatic cyst fluid. J. Mol. Diagn..

[26] Dratwa M, Wysoczańska B, Łacina P, Kubik T, Bogunia-Kubik K (2020). TERT-regulation and roles in cancer formation. Front. Immunol..

[27] Killela PJ (2013). TERT promoter mutations occur frequently in gliomas and a subset of tumors derived from cells with low rates of self-renewal. Proc. Natl. Acad. Sci. USA.

[28] Koorstra JB, Hustinx SR, Offerhaus GJ, Maitra A (2008). Pancreatic carcinogenesis. Pancreatology.

[29] van Heek NT (2002). Telomere shortening is nearly universal in pancreatic intraepithelial neoplasia. Am. J. Pathol..

[30] Matsuda Y (2015). Gradual telomere shortening and increasing chromosomal instability among PanIN grades and normal ductal epithelia with and without cancer in the pancreas. PLoS ONE.

[31] Grant TJ, Hua K, Singh A (2016). Molecular pathogenesis of pancreatic cancer. Prog. Mol. Biol. Transl. Sci..

[32] Zakov S, Kinsella M, Bafna V (2013). An algorithmic approach for breakage-fusion-bridge detection in tumor genomes. Proc. Natl. Acad. Sci. USA.

[33] Hong SM (2011). Telomeres are shortened in acinar-to-ductal metaplasia lesions associated with pancreatic intraepithelial neoplasia but not in isolated acinar-to-ductal metaplasias. Mod. Pathol..

[34] Gomes NM (2011). Comparative biology of mammalian telomeres: Hypotheses on ancestral states and the roles of telomeres in longevity determination. Aging Cell.

[35] Guterres AN, Villanueva J (2020). Targeting telomerase for cancer therapy. Oncogene.

[36] Chiappori AA (2015). A randomized phase II study of the telomerase inhibitor imetelstat as maintenance therapy for advanced non-small-cell lung cancer. Ann. Oncol..

[37] Hesson LB, Cooper WN, Latif F (2007). Evaluation of the 3p21.3 tumour-suppressor gene cluster. Oncogene.

[38] Sharp TV (2008). The chromosome 3p213-encoded gene, LIMD1, is a critical tumor suppressor involved in human lung cancer development. Proc. Natl. Acad. Sci. USA.

[39] Wang K, Ling T, Wu H, Zhang J (2013). Screening of candidate tumor-suppressor genes in 3p21.3 and investigation of the methylation of gene promoters in oral squamous cell carcinoma. Oncol. Rep..

[40] Dammann R (2003). Frequent RASSF1A promoter hypermethylation and K-ras mutations in pancreatic carcinoma. Oncogene.

[41] Amato E (2016). RASSF1 tumor suppressor gene in pancreatic ductal adenocarcinoma: Correlation of expression, chromosomal status and epigenetic changes. BMC Cancer.

[42] Tanaka H, Horikawa I, Barrett JC, Oshimura M (2005). Evidence for inactivation of distinct telomerase repressor genes in different types of human cancers. Int. J. Cancer.

[43] Liu Y (2016). Deletions linked to TP53 loss drive cancer through p53-independent mechanisms. Nature.

[44] Ohira T (2019). PITX1 protein interacts with ZCCHC10 to regulate hTERT mRNA transcription. PLoS ONE.

[45] Kugoh H, Ohira T, Oshimura M (2015). Studies of tumor suppressor genes via chromosome engineering. Cancers.

[46] Kugoh H, Shigenami K, Funaki K, Barrett JC, Oshimura M (2003). Human chromosome 5 carries a putative telomerase repressor gene. Genes Chromosomes Cancer.

