# Supplementary material for: Human chromosome 3p21.3 carries TERT transcriptional regulators in pancreatic cancer
Source: Sci Rep. 2021 Jul 28;11:15355. doi: 10.1038/s41598-021-94711-6 (PMC8319171; doi:10.1038/s41598-021-94711-6)
Supplement: Supplementary file 1 — Supplementary Information. [file 41598_2021_94711_MOESM1_ESM.pdf]

Supplementary Information

Human chromosome 3p21.3 carries *TERT* transcriptional regulators in pancreatic cancer

Takuki Yagyu[1,2], Takahito Ohira[2,3], Ryutaro Shimizu[2,4], Masaki Morimoto[1], Yuki Murakami[1], Takehiko Hanaki[1], Kyoichi Kihara[1], Tomoyuki Matsunaga[1], Manabu Yamamoto[1], Naruo Tokuyasu[1], Teruhisa Sakamoto[1], Yoshiyuki Fujiwara[1], Hiroyuki Kugoh[2,3,*]

[1] Division of Gastrointestinal and Pediatric Surgery, Department of Surgery, School of Medicine, Faculty of Medicine, Tottori University, Yonago, Tottori, Japan.

[2] Department of Molecular and Cellular Biology, Division of Genome and Cellular Function, Tottori University, Yonago, Tottori, Japan

[3] Chromosome Engineering Research Center, Tottori University, Yonago, Tottori, Japan

[4] Division of Urology, Department of Surgery, Faculty of Medicine, Tottori University, Yonago, Tottori, Japan

* Corresponding author: kugoh@tottori-u.ac.jp

Supplementary Figure S1.

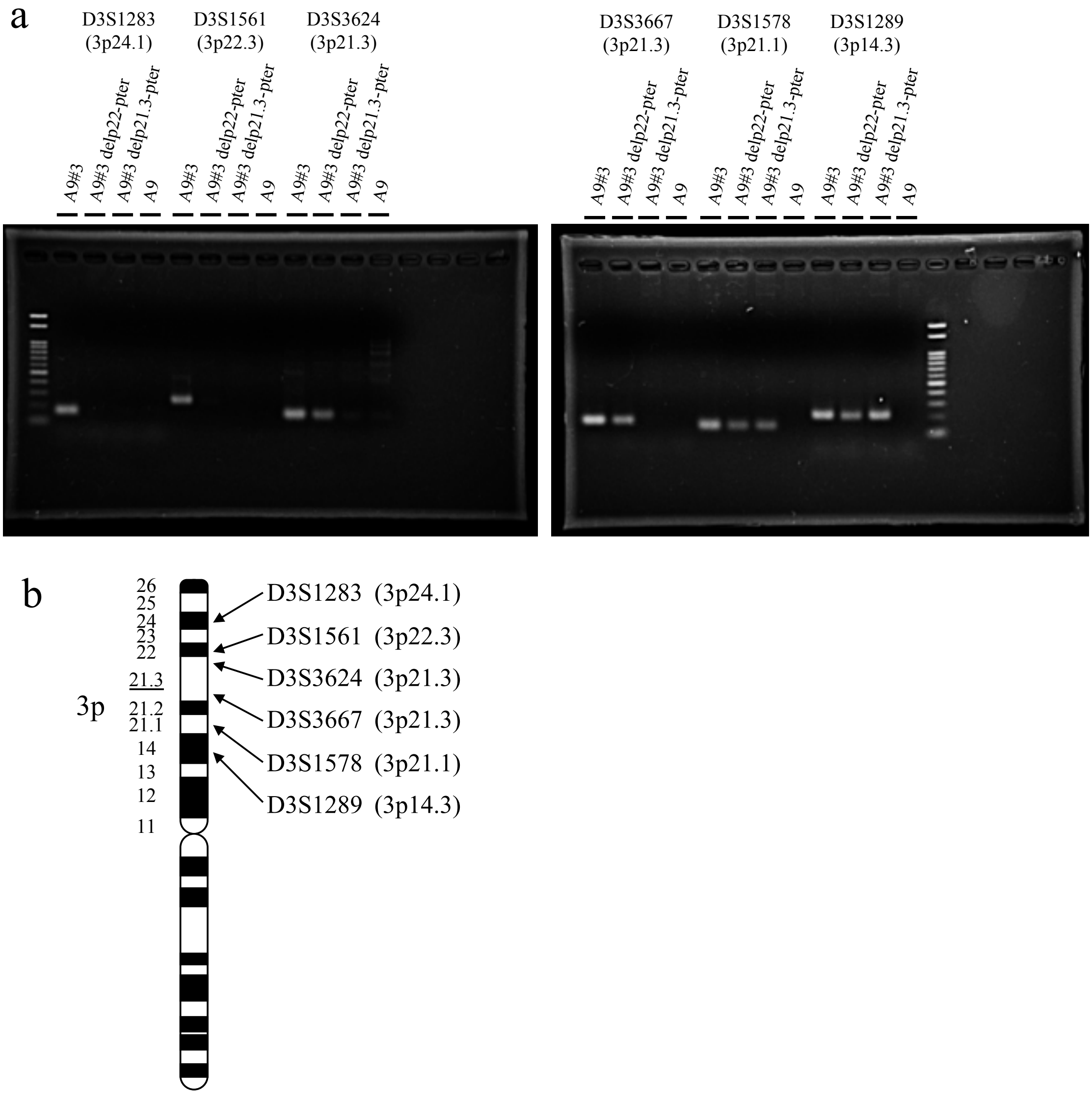

Supplementary Figure S1. Full-length gels of PCR analysis of truncated chromosomes in A9 cells using STS markers for chromosome 3.

(a) Representative results of PCR analysis in A9 microcell hybrid cells as described in Fig 4-a.

(b) Schema of chromosomal regions tagged with each STS marker

Supplementary Figure S2.

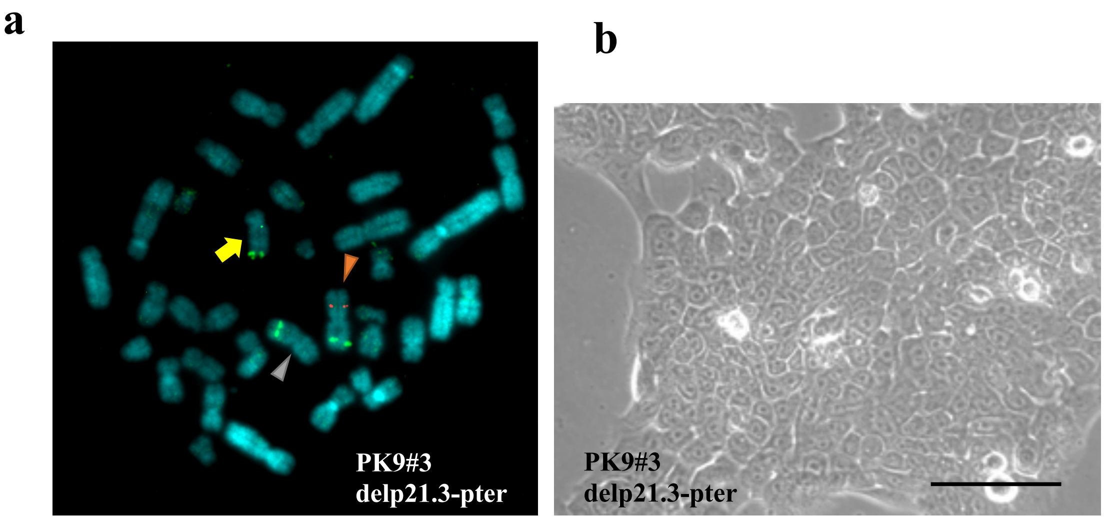

Supplementary Figure S2. PK9 microcell hybrids containing chromosome 3delp21.3-pter were able to establish colonies

(a) Fluorescence in situ hybridization analysis of PK9#3delp21.3-pter. Rhodamine-labeled RP-6 234N4 pPAC4 containing the human chromosome 3p21.3 genomic DNA and FITC-labeled RP11-82C9 BAC that contained the 3q26.2 region were used as probe DNA. The yellow arrow shows a transferred truncated chromosome 3delp21.3-pter. The orange arrowhead indicates a normal human chromosome 3, and the gray arrowhead points out an abnormal chromosome 3. (b) The morphology of PK9#3delp21.3-pter cells. Scale bar 100 μm.

Supplementary Table S1.

	Number of MMCTs	Number of clones established	Ratio of clone acquirement
#3 intact	36 fusions	0	0.0
#4 intact	6 fusions	8	1.25
#3delp21.3-pter	6 fusions	5	0.83

MMCT: Microcell-mediated chromosome transfer
Ratio of clone acquirement: Average number of clones acquired in 1 fusion via MMCT

Supplementary Table S1. Summary of the transfer of human chromosomes 3, 4, and 3delp21.3-pter into PK9 cells
